# Supplementary figures and images for: NUCLEAR FACTOR Y, Subunit C (NF-YC) Transcription Factors Are Positive Regulators of Photomorphogenesis in Arabidopsis thaliana
Source: PLoS Genet. 2016 Sep 29;12(9):e1006333. doi: 10.1371/journal.pgen.1006333 (PMC5042435; doi:10.1371/journal.pgen.1006333)

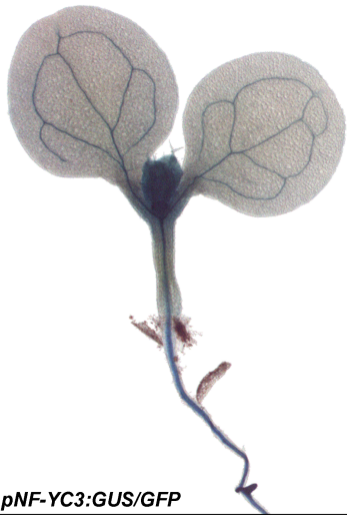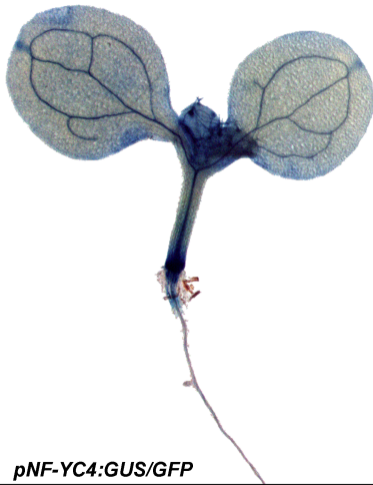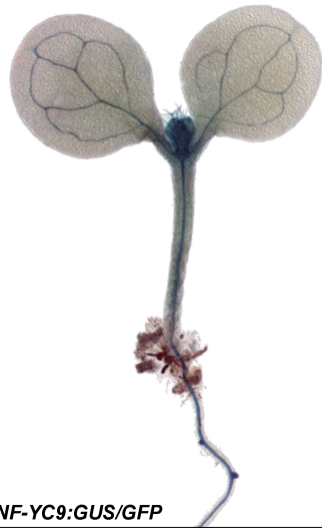

Supplement: S1 Fig — Promoter-GUS fusions for NF-YC3 (left), NF-YC4 (middle), and NF-YC9 (right) were used to analyze expression patterns in 5-day old plants. (PDF) [file pgen.1006333.s001.pdf]

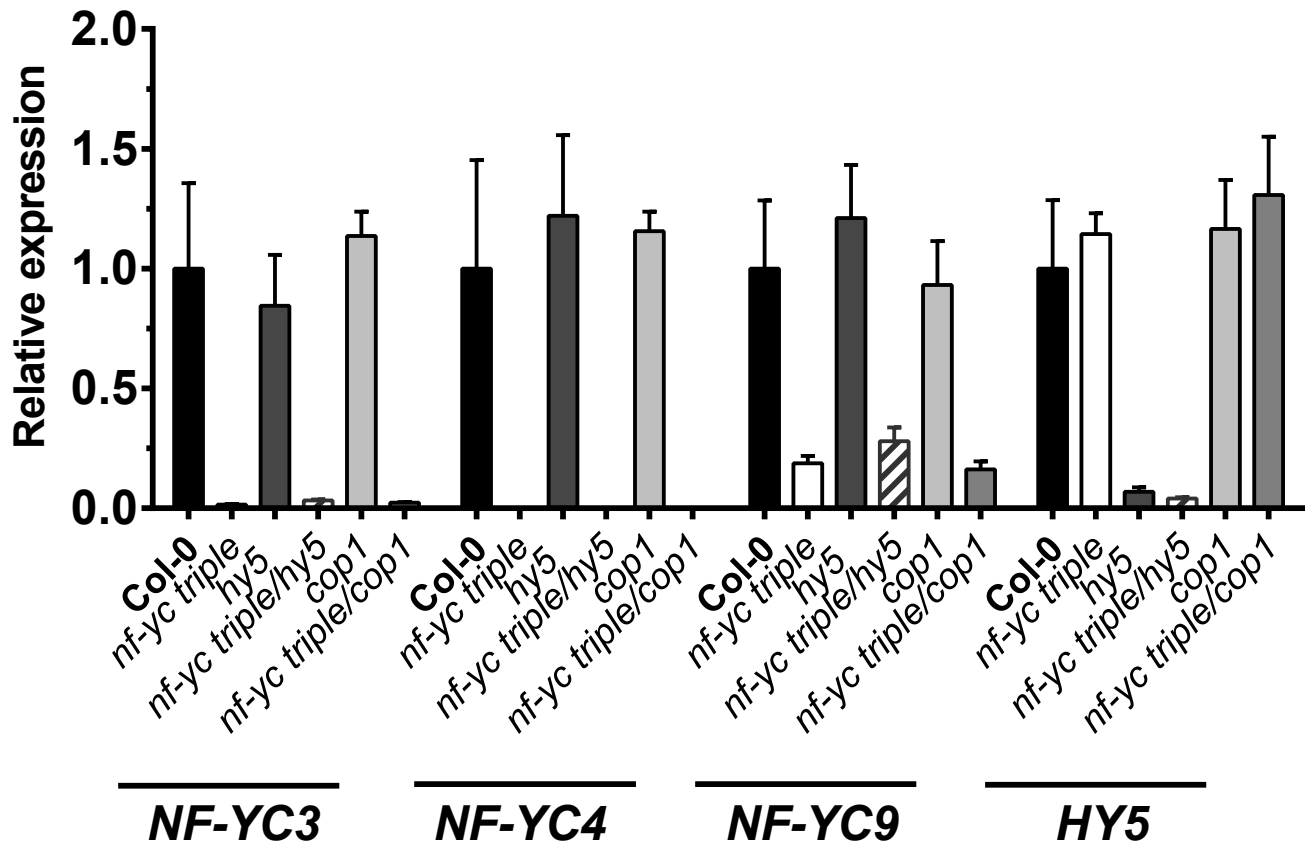

Supplement: S2 Fig — Expression levels of NF-YC3, NF-YC4, NF-YC9, and HY5 were observed in key genotypes used in this study. Note that NF-YC9 is weakly expressed from the nf-yc9-1 allele, as previously reported [51]. Error bars represent SEM. (PDF) [file pgen.1006333.s002.pdf]

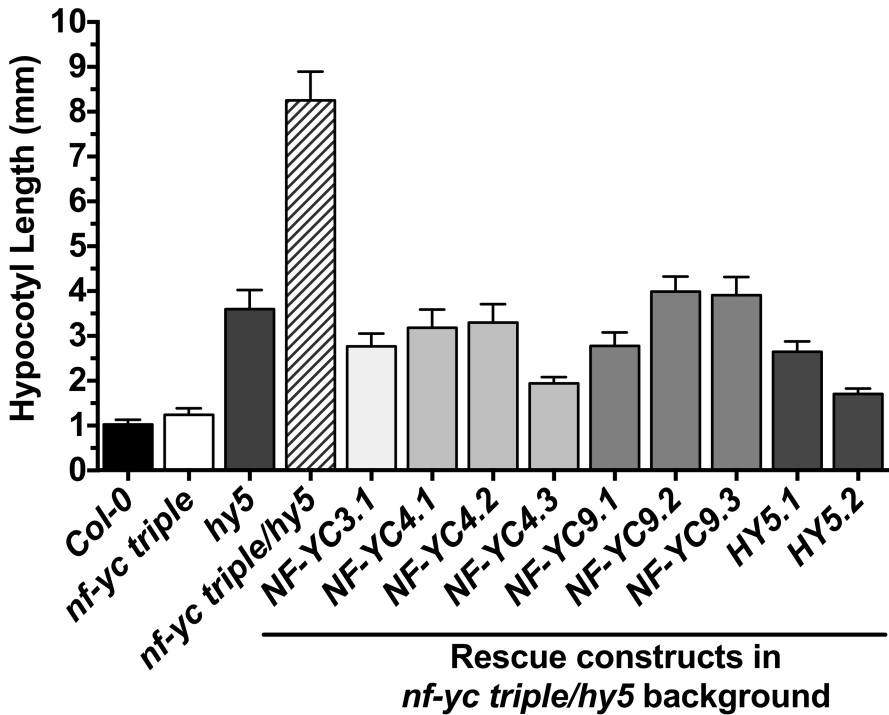

Supplement: S3 Fig — Native-promoter driven NF-YC3, NF-YC4, and NF-YC9, as well as 35S promoter driven HY5, are able to complement the nf-yc triple hy5 mutant hypocotyl phenotype in 5 day old, cWL-grown plants. Error bars represent 95% confidence intervals. (PDF) [file pgen.1006333.s003.pdf]

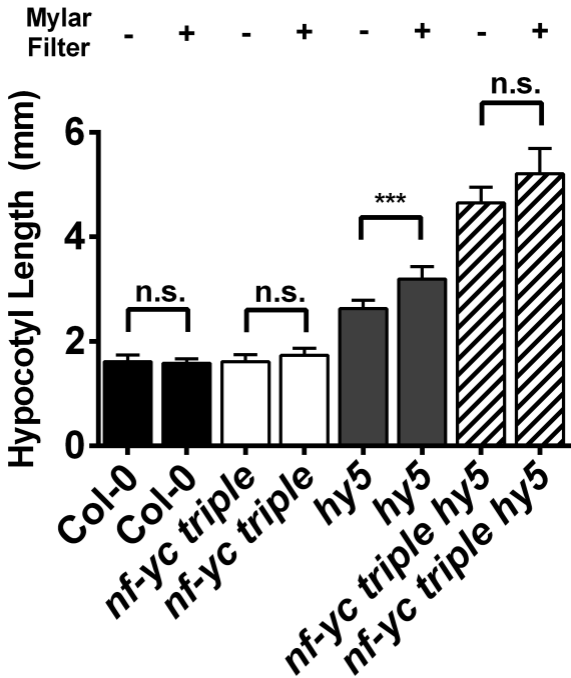

Supplement: S4 Fig — Hypocotyl elongation was assayed in 5 day old, cWL-grown seedlings in the presence or absence of a mylar filter. Error bars represent 95% confidence intervals. Significant differences (and lack thereof) were detected through unpaired t tests (Col-0, nf-yc triple, hy5) and an unpaired t test with Welch’s correction for unequal variances (nf-yc triple hy5). ***, p < 0.01; n.s., not significantly different. (PDF) [file pgen.1006333.s004.pdf]

**Long Day**

**Short Day**

**0**

**4**

**8**

**12**

**16**

**20**

**0**

**4**

**8**

**12**

**16**

**20**

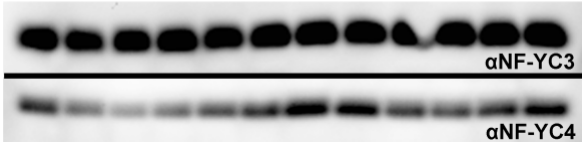

Supplement: S5 Fig — Proteins samples were extracted every four hours from both long day (16hr light, 8hr dark) and short day (8hr light, 16hr dark) grown plants. Proteins were detected with previously described, native antibodies [51]. (PDF) [file pgen.1006333.s005.pdf]
